# Acute Unilateral Oculomotor Nerve Palsy as the Initial Presenting Sign of Nonfunctioning Apoplectic Gonadotroph Adenoma

**DOI:** 10.7759/cureus.8819

**Published:** 2020-06-25

**Authors:** Salem Gaballa, Jane Lindsay, Avan AlJaf, Kyaw M Hlaing, Kashyap Patel

**Affiliations:** 1 Internal Medicine, LewisGale Medical Center, Salem, USA

**Keywords:** pituitary macroadenoma, nonfunctioning adenoma, mass effect, oculomotor nerve palsy, left eye ptosis, transsphenoidal resection, stereotactic radiotherapy

## Abstract

Pituitary macroadenoma usually presents with visual field defects. Oculomotor nerve palsy is a rare presentation, and usually a sign of para-sellar growth and cavernous sinus extension. The oculomotor nerve is more susceptible to laterally transmitted pressure by pituitary mass expansion because of its anatomical location. A slow onset oculomotor nerve palsy results from either gradual compression of the nerve in the sinus wall or direct infiltration of the nerve by the pituitary tumor. We are reporting a unique case of a 68-year-old African American patient who presented to an ophthalmology clinic with left eye complete ptosis, blurry vision, and a progressive headache for a few weeks. He was found to have a nonfunctioning pituitary adenoma (NFPA) that required urgent transnasal transsphenoidal tumor resection. The patient's ocular movements significantly improved a few days postoperatively, and repeated computed tomography (CT) of the head showed complete resection of the pituitary adenoma. The postoperative morning cortisol level was significantly low, confirming the diagnosis of secondary adrenal insufficiency. The patient was treated with corticosteroid replacement therapy.

## Introduction

Most patients with pituitary adenomas present with signs and symptoms of hormone hypersecretion, such as hyperprolactinemia, excess growth hormone, and hypercortisolism. However, 25 to 35% of pituitary adenomas are clinically nonfunctioning, and 70 to 90% of these are gonadotroph adenomas, making them the most common type of pituitary macroadenoma [1]. Pituitary adenoma is one of the most common causes of chiasmal compression and may occur at any adult age, but they are rare in childhood [2]. Ocular motility abnormalities as a result of nonfunctioning pituitary adenoma (NFPA) are not a common presentation. Only a few case reports addressed ocular palsies as a presentation ranging from isolated single-nerve palsy to total ophthalmoplegia. Typically, nonfunctioning adenomas present as macroadenomas that cause neurological symptoms due to intracranial mass effects, since hormonal inactivity leads to a delay in diagnosis compared with functioning pituitary adenomas [3]. If pituitary adenomas are not treated, vision will continue to deteriorate, and blindness might occur. A relationship exists between the severity of visual impairment and tumor size [3].

## Case presentation

**Figure 1:**
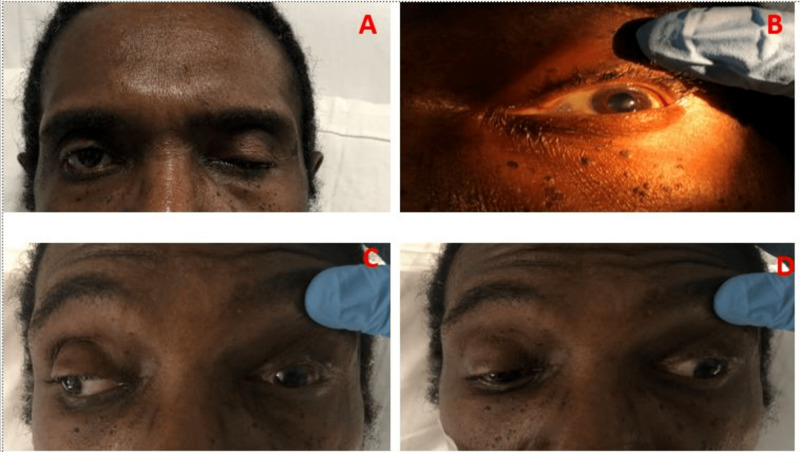
A) showing complete left eye ptosis because of the affected levator palpebrae superioris muscle. B) showing left pupil mydriasis due to the involved parasympathetic fibers that innervate the muscles responsible for pupil constriction (sphincter pupillae). C) showing inability to adduct the left eye while looking to the right due to the affected medial rectus of the left eye that is innervated by the oculomotor nerve. D) showing the left eye displaced outward “exotropia” because the lateral rectus (innervated by the sixth cranial nerve) maintains muscle tone in comparison to the paralyzed medial rectus, and displaced downward “hypotropia” because the superior oblique (innervated by the fourth cranial or trochlear nerve), is unantagonized by the paralyzed superior rectus, inferior rectus and inferior oblique.

A 68-year-old African American male with a past medical history of hypertension and benign prostatic hyperplasia was brought to the emergency department as a transfer from an ophthalmology clinic for acute onset left eye abnormality. The patient reported rapidly declining eye movements (was unable to open his left eye as well), diplopia, and blurry vision. The patient endorsed an associated throbbing headache, decreased energy, and depressed mood. The patient denied any heat or cold intolerance, weight changes, diarrhea or constipation, and any changes in libido. The physical examination showed complete left eye ptosis, mydriasis, and limited adduction. His left eye was deviated downward and outward (Figure 1). His neurological exam was unremarkable.

**Figure 2:**
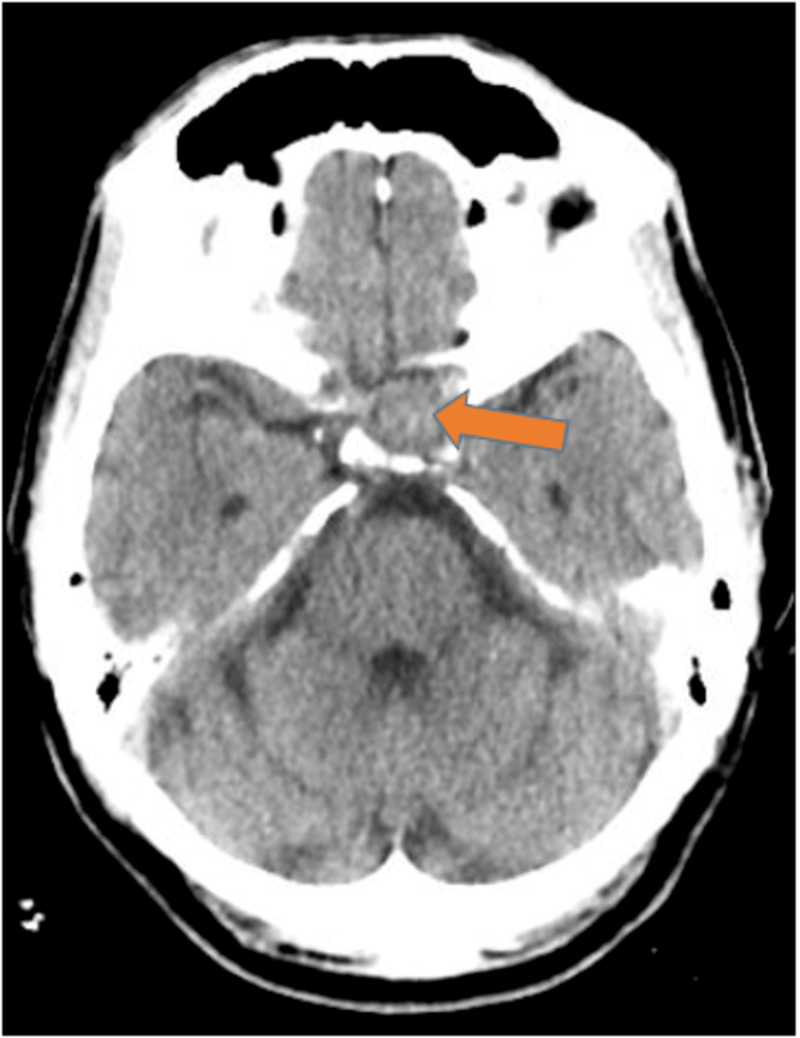
CT head with IV contrast showed a large 2.2 x 1.9 cm mass in the region of the pituitary gland splaying the vascular system as shown by the orange arrow.

**Figure 3:**
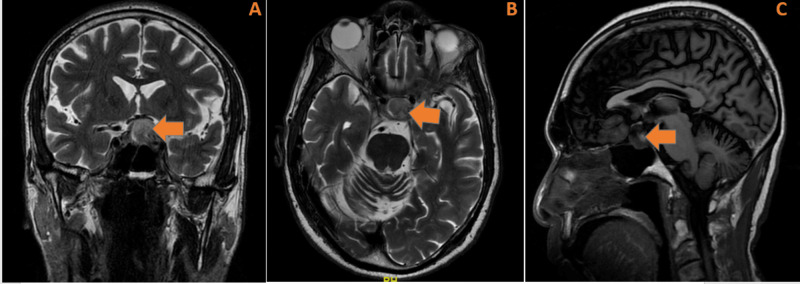
MRI images in different views. A) T1-weighted-fluid-attenuated inversion recovery MRI (coronal view), B) T2-weighted-FSE (axial view), C) T1-weighted-fluid-attenuated inversion recovery (sagittal view), showing heterogeneously enhancing mass within the sella turcica measuring 1.6 x 1.9 x 1.7 cm as shown by orange arrows causing elevation of the optic chiasma and laterally extending into the cavernous sinus. FSE: Fast Spin Echo

**Figure 4:**
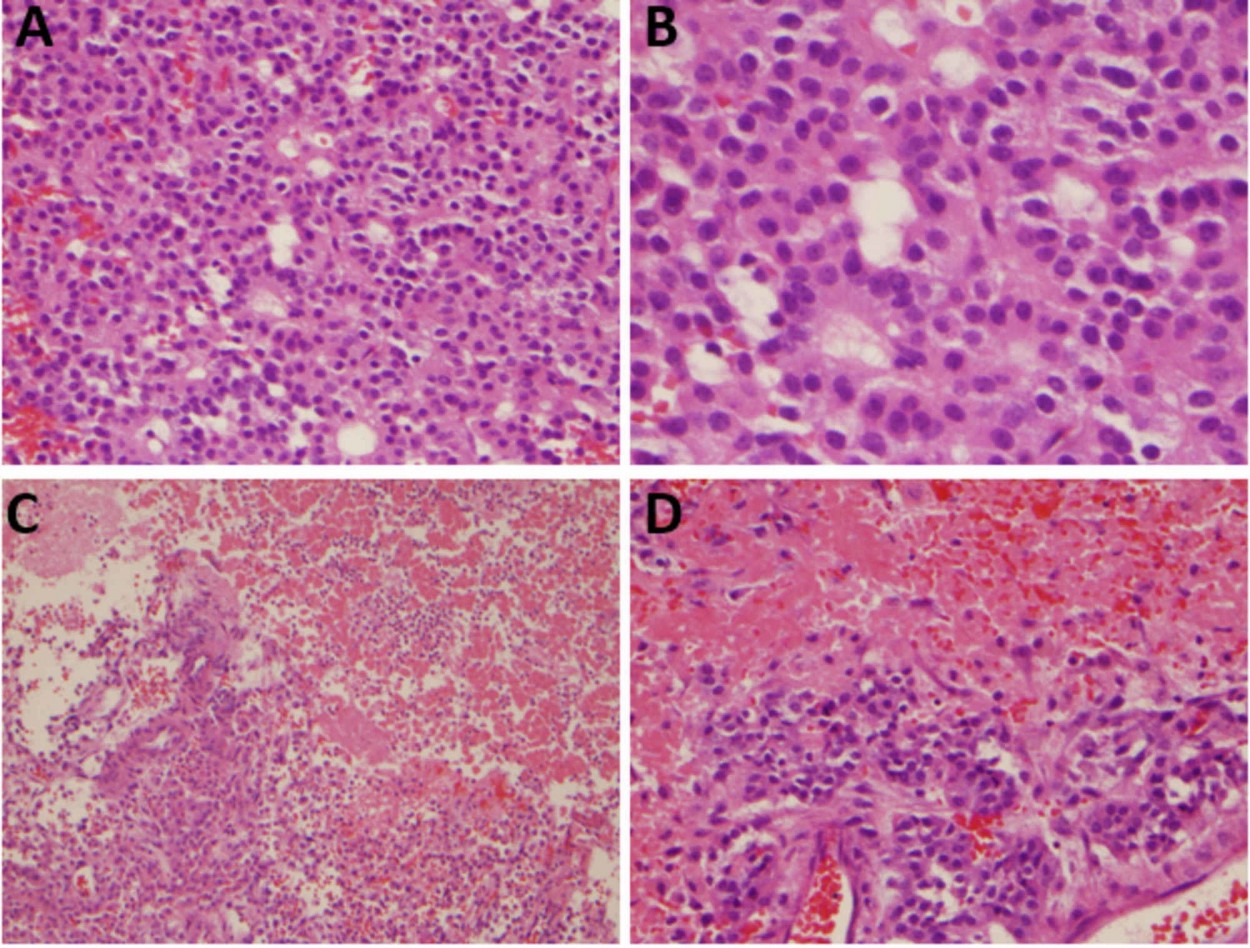
A) Hematoxylin and eosin (H&E) stained section showing a pituitary adenoma composed of monomorphous population of medium-sized cells with pale eosinophilic to clear cytoplasm and central round nuclei. The tumor cells are arranged in perivascular or papillary arrangements [H&E; 20x original magnification]. B) A high magnification of the adenoma with details of the homogeneous population of epithelial-like cells with nuclei with dispersed chromatin [H&E; 40x original magnification]. C) In several areas of the specimen, necrosis of the tumor was observed with ghosts of the tumor cells and moderate infiltration of neutrophils and hemorrhage (upper right) [H&E; 20x magnification]. D) A higher magnification view of area of transition between viable adenoma (bottom) and necrotic tumor (upper) [H&E; 40x magnification].

**Figure 5:**
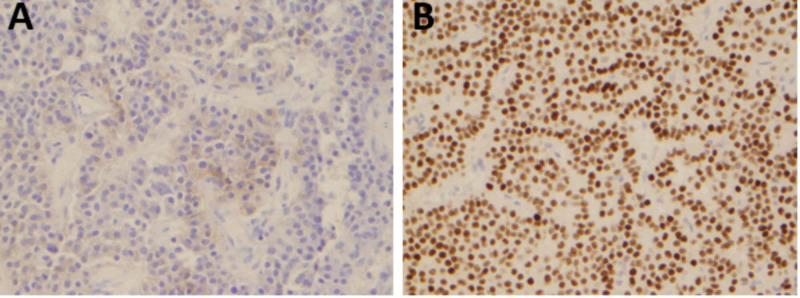
A) Immunohistochemistry for pituitary hormones demonstrated focal positivity of the tumor cells for Beta-FSH. [Beta-FSH IHC with diaminobenzidine chromogen (golden brown) and hematoxylin counterstain; 40x original magnification]. B) The tumor shows diffuse and strong nuclear immunoreactivity for the gonadotroph-lineage transcription factor SF-1 confirming gonadotroph differentiation. [SF-1 IHC; 40x original magnification]. Beta-FSH: beta subunit-follicle stimulating hormone Beta-FSH IHC: beta subunit-follicle stimulating hormone immunohistochemistry SF-1 IHC: steroidogenic factor-1 immunohistochemistry

In the emergency department, complete blood count (CBC) and comprehensive metabolic panel (CMP) were within normal limits. Full endocrine function tests revealed a mildly elevated luteinizing hormone (LH) (10.3 IU/L, normal range 1.24-7.8 IU/L), follicle-stimulating hormone (FSH) (15.3 IU/L, normal range 1.5-12.4 IU/L) and insulin growth factor-1 (263 ng/ml, normal range 47-192 ng/ml). Prolactin, adrenocorticotropic hormone cortisol (ACTH), thyroid-stimulating hormone (TSH), and free thyroxin (T4) were within the normal limits. CT angiography (CTA) of the head with intravenous contrast did not reveal any evidence of intracranial aneurysm or evidence of hemodynamically significant stenosis but showed a large 2.2 x 1.9 cm mass in the region of the pituitary gland splaying the vascular system as shown in Figure 2. Urgent magnetic resonance imaging (MRI) of the brain with and without intravenous contrast showed a heterogeneously enhancing mass within the sella turcica measuring about 1.6 x 1.9 x 1.7 cm, as shown in Figure 3. An urgent neurosurgical evaluation was placed, and the following day he underwent an emergent transnasal transsphenoidal pituitary resection by an experienced neurosurgeon. The procedure was complicated by cerebrospinal fluid (CSF) leak and required a nasoseptal flap. Tissue pathology revealed a diagnosis of apoplectic gonadotroph adenoma, immunoreactive for FSH, and the transcription factor SF-1 that drives gonadotroph differentiation (Figures 4, 5).

**Figure 6:**
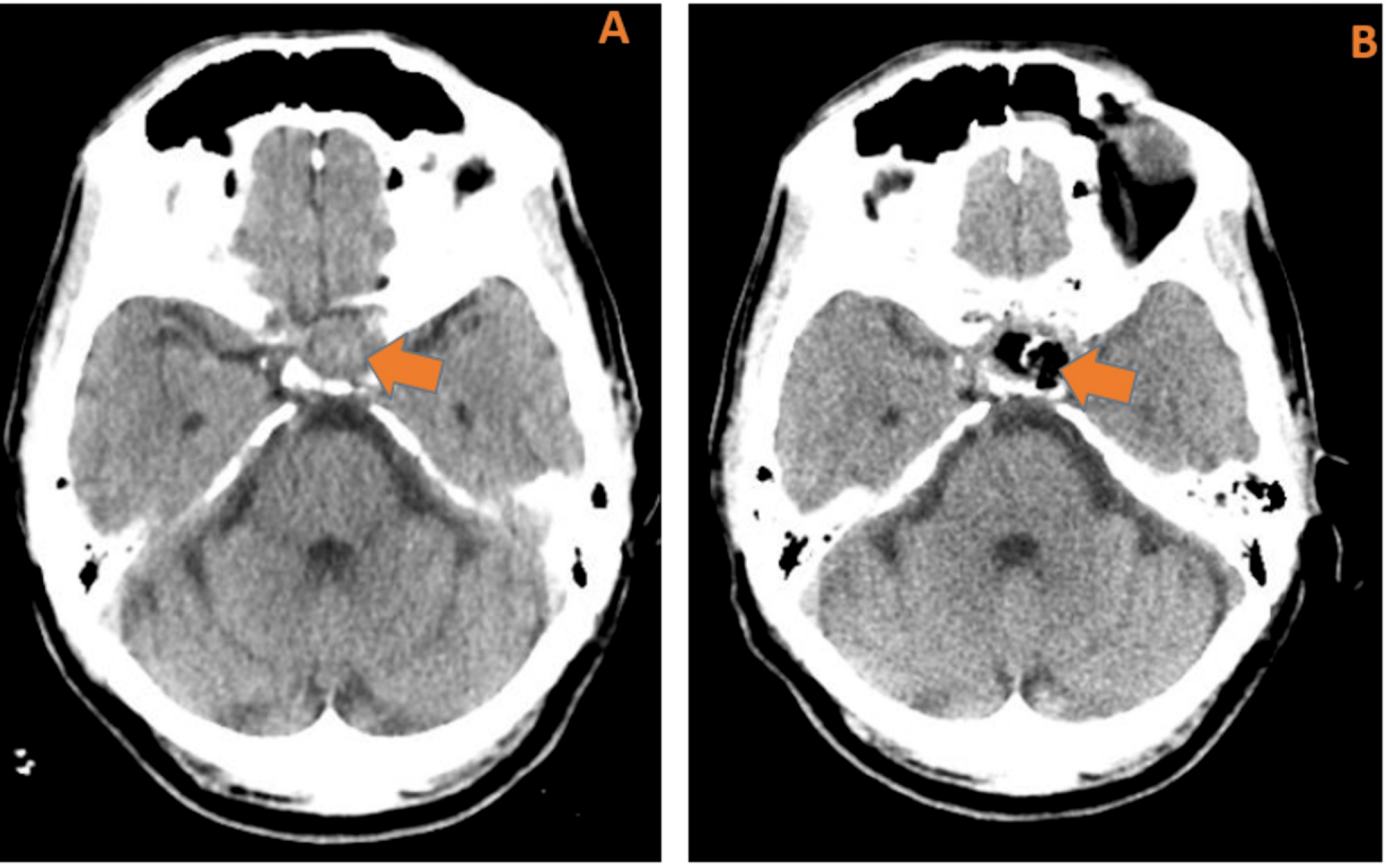
As a comparison with CT head preoperatively (A), CT head without IV contrast, postoperatively (B) showed complete resection of pituitary macroadenoma without a significant residual tumor remaining as shown by orange arrow and associated with small amount of expected left-sided pneumocephalus.

**Figure 7:**
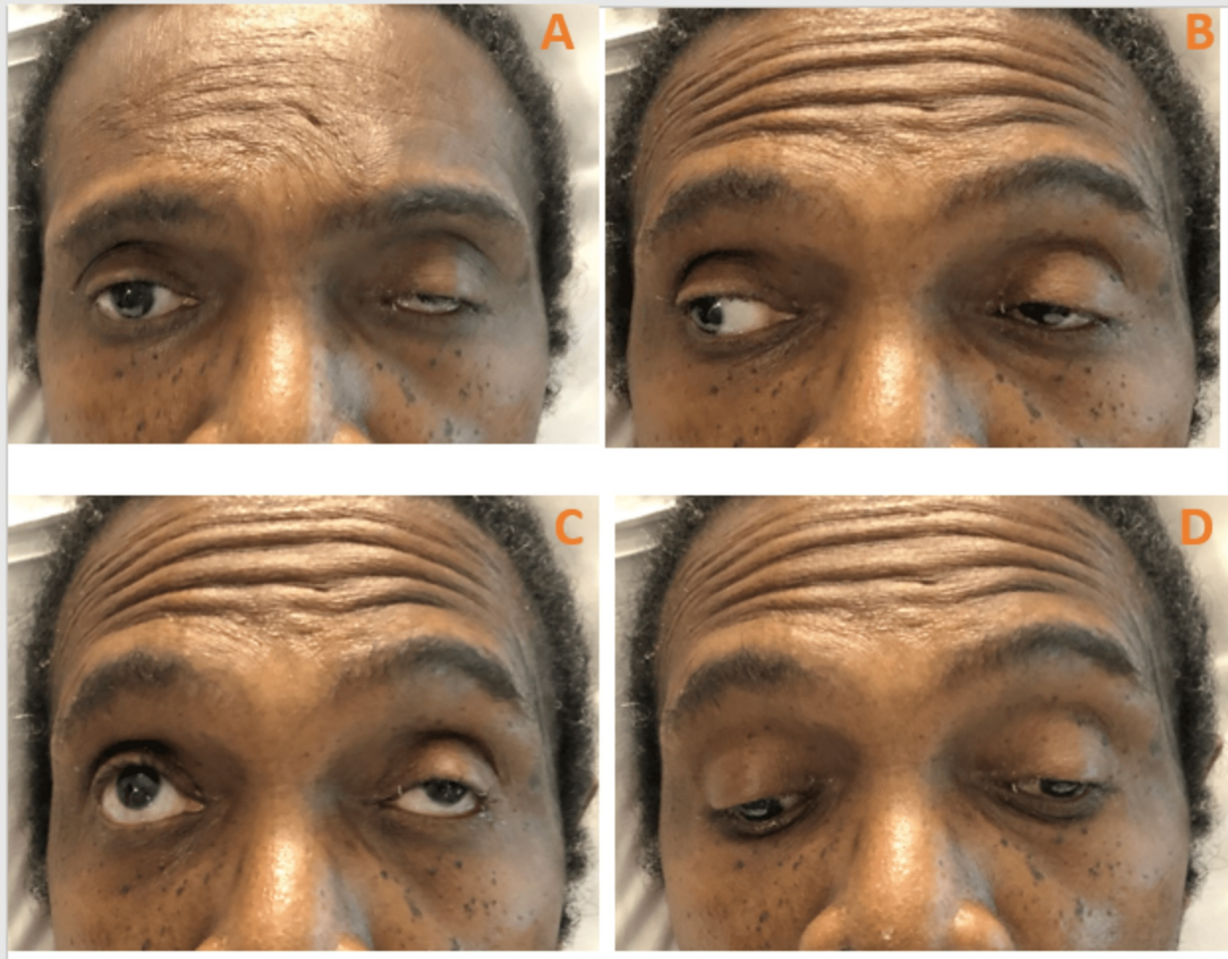
Postoperative day 6 showing improvement of the left eye movements: A) showing a partial improvement of the left eye ptosis. B) showing improvement of the left eye adduction while looking to the right. C) showing improvement of the left eye while looking upwards, and no longer displaced downward and outward. D) showing improvement of the left eye while looking downwards.

Endocrinology was consulted and ordered an early morning cortisone level, which was low (3.5 mcg/dL, AM normal range 5.2-22.5 mcg/dL), confirming the diagnosis of secondary adrenal insufficiency. Free thyroxine and serum testosterone were within the normal limits. Endocrinology recommended treating the secondary adrenal insufficiency with a one-time dose of 100 mg intravenous hydrocortisone, then continued on a daily replacement of 25 mg intravenous hydrocortisone every 6 hours. Afterward, he was transitioned to PO hydrocortisone, 20 mg every morning, and 10 mg every evening. Postoperative CT head without intravenous contrast (Figure 6) showed complete resection of pituitary macroadenoma without a significant residual tumor remaining. There was a small amount of expected left-sided pneumocephalus. On postoperative day 6, the left eye ptosis and movement abnormalities started to improve, as shown in Figure 7. The patient was discharged home on oral hydrocortisone 20 mg in the morning and 10 mg at night and was scheduled to follow up outpatient with endocrinology and neurosurgery.

## Discussion

Pituitary adenomas are categorized by their cell of origin (lactotroph, gonadotroph, somatotroph, corticotroph, and thyrotroph) and their size (microadenomas <1 cm, macroadenomas ≥1 cm). Most adenomas (65 to 70%) secrete an excess amount of hormone, including prolactin, growth hormone (GH), corticotropin (ACTH), or TSH. The remainder of pituitary adenomas (25 to 35%) are clinically nonfunctioning or "silent." Of these, 70 to 90% are gonadotroph adenomas. There are also clinically nonfunctioning somatotroph, lactotroph, and corticotroph adenomas, although these are less common [4]. The majority of gonadotroph adenomas are clinically "silent" and difficult to identify because they are poorly differentiated and produce and secrete hormones inefficiently.

The gonadotropins, LH and FSH, consist of a common alpha subunit and a unique beta subunit. TSH and human chorionic gonadotropin (hCG) also consist of the common alpha subunit and a unique beta subunit. The hormones secreted by gonadotroph adenomas in order of decreasing frequency include FSH, FSH-beta, alpha subunit, LH, and LH-beta [5]. The pathogenesis of gonadotroph adenomas, like other pituitary adenomas, appears to be true clonal neoplasms, but the mutations that cause them are not known. A genetic analysis reported the overexpression of the pituitary tumor transforming gene, Ki-67, and FGF-R, and the underexpression of the maternally expressed gene 3 (MEG3) [6].

Nonfunctioning pituitary adenomas (NFPA), including gonadotroph adenomas, are slow-growing and difficult to recognize clinically until they are large enough to cause symptoms due to a mass effect. The most common presenting complaints are: a) neurologic symptoms - such as visual disturbances and to a lesser extent headaches, b) incidental pituitary mass discovered during brain imaging for reasons other than pituitary disease, c) hypopituitarism due to compression of normal pituitary tissue by the adenoma. In a published study by Young et al. the clinical symptoms and factors leading to a diagnosis of pituitary tumor included: visual loss (43%), symptoms of hypopituitarism (22%), no symptoms (incidental diagnosis, 17%), headache (8%), and a combination of visual loss, hypopituitarism, and headache (10%) [7].

The suprasellar extension of NFA compresses the optic chasm, causing visual impairments, which is the most common symptom that leads a patient to seek medical attention [8]. Per Molitch study that included 1719 patients with clinically nonfunctioning pituitary adenomas, visual field disturbances were present in 798 (46%) [9]. Diplopia is caused by the lateral extension of NFA that results in oculomotor nerve compression, which occurs in up to 10 to 15% of patients with large NFA [9]. Diffuse headache is the second most common neurologic symptom, which occurs in 30 to 40% of patients with NFA. Suggested mechanism by which NFA cause headaches is due to sellar expansion, causing increased intracranial pressure and possibly a stretch of dura mater [9]. Other less common neurologic symptoms include cerebrospinal fluid (CSF) rhinorrhea due to the inferior extension of the adenoma and pituitary apoplexy (sudden hemorrhage into a pituitary macroadenoma) that leads to excruciating headache and visual impairment.

Clinically NFPA often presents with evidence of hypopituitarism and rarely can present with hormonal hypersecretion causing a clinical syndrome such as ovarian hyperstimulation or precocious puberty [9]. NFPA causes hormonal insufficiency by compressing the functioning nonadenomatous cells and the pituitary stalk, which results in decreased availability of hypothalamic stimulatory hormones. The most common clinical hormone deficiency is the impaired secretion of gonadotropins resulting in hypogonadism. In a published study by Ferrante et al. that included 295 patients with nonfunctioning pituitary adenomas, 61 of 161 men (38%) had low serum gonadotropins, resulting in low serum testosterone, decreased libido, and erectile dysfunction. In the same report, 33% of the women of reproductive age had menstrual cycle disorders [10]. The majority of hypopituitarism may be detected biochemically in patients with clinically NFA. Per Drummond et al., in a review of 1719 patients, 993 (58%) had laboratory evidence of pituitary hormone deficiency. GH deficiency was 87% (220 of 252 tested), LH/FSH deficiency (hypogonadotropic hypogonadism; 1216 of 1699 patients tested), was 72%. ACTH deficiency (secondary adrenal insufficiency; 514 of 1699) was 30%. TSH deficiency (central hypothyroidism; 402 of 1699) was 24% [11].

Although gonadotroph adenomas are considered to be "nonfunctioning," most do produce intact gonadotropins or their subunits. However, these adenomas are typically poorly differentiated and inefficient producers/secretors and do not raise serum gonadotropin concentrations. Thus, they are usually clinically "silent" and cannot be distinguished from other clinically nonfunctioning adenomas until immunohistochemistry is performed after pituitary surgery [12]. However, approximately 35% of gonadotroph adenomas secrete enough LH or FSH to raise serum gonadotropin levels, but it does not result in a clinical syndrome. It is reported in rare case reports that gonadotroph adenomas cause ovarian hyperstimulation in premenopausal women and precocious puberty in prepuberty boys [13]. NFA can be accompanied by hyperprolactinemia as a result of the compression of the pituitary stalk and the obstruction of the normal inhibitory hypothalamic influence on the prolactin-producing cells, thus resulting in slightly elevated serum prolactin concentrations (usually <100 ng/mL but sometimes as high as 200 ng/mL). Rarely, gonadotroph adenomas co-secrete prolactin and gonadotropins [14]. It is recommended that patients with incidental adenomas be screened for hypersecretion and hyposecretion. In the absence of hypersecretion, hypopituitarism, or visual field defects, it is recommended to do periodic monitoring by MRI for possible enlargement [14].

CT and MRI have revolutionized pituitary imaging. Unlike CT, MRI can be performed repeatedly without risk of excessive exposure to X-rays and shows surrounding soft tissue structures such as the pituitary stalk and optic chiasm. The sensitivity of the detection of surgically proved microadenomas approaches 100% with MRI compared with 50% with CT, but 17% of pituitary lesions causing Cushing's disease are too small or diffuse to be reliably identified by either technique. Another advantage of MRI is the ability to distinguish other sellar abnormalities, such as carotid artery aneurysm or hemorrhage into a pituitary tumor. Occasionally, the apparent NFPA may represent meningioma, craniopharyngioma, granulomatous diseases, gliomas, chordomas, Rathke's cleft cyst, or metastatic disease [15].

The goals of treatment in patients with gonadotroph adenomas or any other NFPA include a relief of visual impairment or other neurologic symptoms, removal of as much of the adenoma as possible to avoid recurrence, and replacement of hormonal deficiencies. Standard first-line therapy of NFPA (greater than 1 cm) is transsphenoidal surgery, which should be promptly instituted for those with impaired vision and considered for those at high risk for loss of vision (marked suprasellar extension). For patients who have a suprasellar extension but do not have neurologic symptoms, we should discuss the risks of surgery versus the risks of waiting. Gonadotroph adenomas that are asymptomatic and not an immediate threat to vision may not require immediate surgery [16]. Although transsphenoidal surgery has a low mortality rate and acceptable surgical complications, the accomplishment of total or near-total resection can be challenging and varies in different cases, ranging from 20% to 80% [16].

Transsphenoidal surgery improves vision in approximately 80% of patients; improvement can be seen in the first few days after surgery. If the vision was abnormal before surgery, it should be reevaluated a month or two afterward and less often until no further change occurs [17]. Unlike the improvement in vision, improvement in pituitary function is less likely. In a systemic review and meta-analysis of the surgical outcomes of NFA in patients with preoperative pituitary hormone abnormalities, 30% of patients experienced an improvement in some hormone deficiency. However, most patients are left with long-term deficiencies that need replacement [17]. The reported complications of transsphenoidal surgery are less than 5% and include cerebrospinal fluid leakage, fistula, meningitis, and new visual field defects. The risk of complications is inversely proportional to the experience of the surgeon in performing transsphenoidal surgery [17].

Pituitary MRI is recommended four to six weeks after discharge from the hospital to evaluate the amount of residual adenoma, visual function (by acuity and visual fields), and hormonal function of the nonadenomatous pituitary, regardless of whether it was normal or abnormal prior to surgery. This hormonal evaluation should include measurements of T4, early morning serum cortisol level, serum testosterone in men or serum estradiol in premenopausal women, and 24-hour urine volume if the patient has significant nocturia [18]. If there is no or little discernible residual adenoma tissue confirmed by pituitary MRI following the surgery, the patient should be monitored by MRI and hormonally, initially at six-month intervals. If there is considerable residual adenomatous tissue, radiation should be administered. Long-term monitoring is necessary because the risk of adenoma regrowth is significant, particularly after surgery alone [18].

Radiation therapy (RT) has shown to be effective as an adjunct to surgical resection in cases of postoperative residual tumor or recurrence. The decision to recommend radiation is based on the amount and location of the residual tissue by MRI, the aggressiveness of the adenoma, and the patient's age and general health. Per Wahba et al., conventional external RT up to 54 Gy is safe and effective in controlling nonfunctioning pituitary macroadenoma with tolerable and acceptable morbidity [19]. Stereotactic techniques such as stereotactic radiosurgery or fractionated stereotactic radiotherapy have more localized irradiation and fewer long-term side-effects [19]. Both techniques provide excellent tumor control in patients with NFPAs, ranging from 85% to 95% at five to 10 years [19].

Oculomotor nerve palsy is not as common in NFPA as the visual disturbances or hormonal deficiencies. In a clinical study done by Chuang et al., a total of 23 patients with pituitary adenomas presenting with oculomotor nerve palsy were treated immediately with glucocorticoid therapy. Elective transsphenoidal surgery was used for decompression and histopathological confirmation. The study concluded that oculomotor nerve palsy usually occurs in patients with apoplectic adenomas, especially those with hemorrhage. Early treatment, pupil-sparing, and minor oculomotor symptoms are factors indicating a good recovery [20].

## Conclusions

One-third of pituitary adenomas are clinically NFPA, and the majority of them are gonadotroph adenomas. Oculomotor nerve palsy may clinically raise the possibility of pituitary adenomas. Diagnosis is usually clear after examination and from the patient's history and can be confirmed with a scan of the pituitary (by CT or preferably MRI) and the appropriate hormone evaluation tests. First-line standard treatment for NFPA that causes hormonal hypersecretion or hyposecretion and neurological symptoms is usually transsphenoidal surgery. If there is no or little discernible residual adenoma tissue by MRI following surgery, the patient should be monitored by MRI and hormonally, initially at six-month intervals. Standard radiotherapy halves the rate of tumor recurrence but is now less commonly given as an adjunct to surgery because of reports of adverse effects.
